# An Efficient Sodium Borate‐Based Electrolyte Additive to Activate Sulfur Conversion and Stabilize Calcium Anodes in Calcium−Sulfur Batteries

**DOI:** 10.1002/smll.74655

**Published:** 2026-07-21

**Authors:** Sebahat Topal, Ping Feng, Marco Kögel, Sibylle Riedel, Joachim Haecker, Maryam Nojabaee, Thomas Diemant, Darius Hübner, Jannik C. Meyer, Timo Jacob, Maximilian Fichtner, Zhirong Zhao‐Karger

**Affiliations:** ^1^ Helmholtz Institute Ulm (HIU) Electrochemical Energy Storage Ulm Germany; ^2^ NMI Natural and Medical Sciences Institute at the University of Tübingen Reutlingen Germany; ^3^ Institute of Applied Physics Eberhard Karls University of Tübingen Tübingen Germany; ^4^ Deutsches Zentrum für Luft‐ und Raumfahrt e. V. (DLR) German Aerospace Center Institute of Engineering Thermodynamics, Electrochemical Energy Technology Stuttgart Germany; ^5^ Institute of Electrochemistry Ulm University Ulm Germany; ^6^ Karlsruhe Institute of Technology (KIT) Karlsruhe Germany; ^7^ Institute of Nanotechnology, Karlsruhe Institute of Technology (KIT) Eggenstein‐Leopoldshafen Germany

**Keywords:** calcium anodes, electrolyte additives, fluorinated borate electrolytes, rechargeable calcium–sulfur batteries

## Abstract

Calcium−sulfur (Ca−S) batteries are considered promising candidates for large‐scale energy storage owing to their high theoretical energy density, the natural abundance of both calcium and sulfur, and their inherent cost‐effectiveness and sustainability. A room‐temperature, reversible Ca–S battery has previously been demonstrated as a proof of concept using the calcium tetrakis(hexafluoroisopropyloxy)borate (Ca[B(hfip)_4_]_2_) electrolyte; however, it still suffers from limited cycling stability and severe Ca anode passivation. Herein, we report the improvement of the Ca−S battery performance using a sodium borate‐modified electrolyte. We demonstrate that the introduction of Na^+^ can enhance the reversibility of sulfur redox chemistry and the Ca plating/stripping at the anode simultaneously. The Ca−S coin cells with a S/Ketjenblack/Polyaniline cathode and Ca metal anode deliver a high initial discharge capacity of 867 mAh g^−1^ at 0.1C (1C = 1672 mA g^−1^) and maintain a stable discharge voltage of around 2.2 V and a capacity of 96 mAh g^−1^ after 80 cycles. The modified electrolyte also enables stable operation of a Ca−S pouch cell for 50 cycles with a discharge capacity of 80 mAh g^−1^ at 0.1C. This work presents an effective electrolyte modification approach for advancing Ca batteries.

## Graphical abstract

Calcium−sulfur (Ca−S) batteries are promising candidates for sustainable energy storage; yet their development is limited by the lack of efficient electrolytes. Here, a sodium‐ion modified Ca[B(hfip)_4_]_2_ electrolyte enables reversible room‐temperature Ca−S batteries by simultaneously improving Ca plating/stripping stability and reversible sulfur conversion, providing an effective electrolyte‐engineering strategy for advanced Ca batteries.

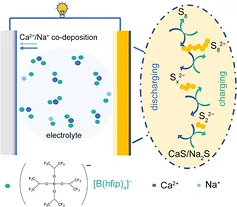

## 1 Introduction

Post‐lithium storage technologies play a key role in achieving clean, sustainable, environmentally friendly, cost‐effective, and safe energy solutions [1, 2]. Among various post‐lithium systems (e.g., Na, Mg, and Ca), calcium (Ca) batteries employing metallic Ca anodes have emerged as one of the most promising candidates due to the intrinsic advantages of calcium metal [3, 4, 5, 6, 7]. Calcium is the fifth most abundant element in the Earth's crust (Ca: 3.45 wt.% vs. Li: 0.002 wt.%), with resources evenly distributed worldwide [7]. It possesses a low redox potential (−2.87 V vs. standard hydrogen electrode, SHE), comparable to that of Li^+^/Li (−3.04 V vs. SHE) and 0.5 V lower than that of Mg^2+^/Mg (−2.37 V vs. SHE). Moreover, Ca metal exhibits a high theoretical capacity of 2073 mAh cm^−3^ and 1337 mAh g^−1^ [7]. Thus, the theoretical voltage and energy density of Ca batteries are comparable to those of Li‐based systems. Sulfur is a low‐cost and sustainable cathode material, and it offers a high theoretical capacity of 1672 mAh g^−1^. When coupled with a Ca metal anode, Ca−S batteries can deliver a theoretical cell voltage of 2.47 V and an energy density of 3202 Wh L^−1^ and 1835 Wh kg^−1^, based on a two‐electron conversion reaction with the formation of CaS (Ca + S −> CaS, Δ_r_G^0^ = −477.4 kJ mol^−1^) [8]. This value is comparable to Mg−S systems, yet exceeds that of Li−S batteries (2800 Wh L^−1^), making Ca−S batteries an excellent option for grid‐scale energy storage systems [9]. Unfortunately, the sluggish charge transfer kinetics of the S redox reaction lead to incomplete conversion of active materials and cause limited reaction reversibility [10]. The incompatibility between electrolyte and Ca metal often triggers severe side reactions and surface passivation on Ca, which in turn leads to irreversible anode redox reaction at the Ca anodes and challenges the development of reversible Ca−S batteries at room temperature [11].

To achieve reversible Ca batteries, significant efforts have focused on developing suitable electrolytes. In conventional aprotic systems, such as Ca(BF_4_)_2_ in ethylene carbonate/diethyl carbonate [3], Ca(ClO_4_)_2_ in acetonitrile [12], and Ca(CF_3_SO_3_)_2_ in glyme [13], irreversible parasitic reactions between Ca metal and the electrolyte lead to the formation of nonconductive surface layers (containing e.g., CaF_2_, Ca(OH)_2_, CaCO_3_), ultimately causing cell failure. With the advent of a new class of calcium electrolytes featuring weakly coordinating anions, calcium tetrakis(hexafluoroisopropoxy)borate (Ca[B(hfip)_4_]_2_) emerged as the first efficient room‐temperature electrolyte for nonaqueous calcium batteries [11]. Ca−S batteries employing Ca[B(hfip)_4_]_2_ as the electrolyte exhibited a high initial capacity of approximately 760 mAh g^−1^ and retained a capacity of about 120 mAh g^−1^ after 15 cycles [9]. However, the dissolution of the polysulfides and partial formation of a passivation layer on the Ca anode impaired cycle life. Hybrid‐ion electrolytes, such as Ca(CF_3_SO_3_)_2_−LiCF_3_SO_3_ in tetraglyme, delivered an initial discharge capacity of approximately 800 mAh g^−1^ in Ca−S batteries and retained a capacity of about 300 mAh g^−1^ after 20 cycles. Nevertheless, the observed voltage plateau (∼1.1 V at 0.1C) remained significantly below the theoretical voltage associated with CaS formation [13]. Building on the strategy of incorporating Li^+^ ions into the electrolyte to improve the cycling stability of Ca−S batteries, a LiPF_6_/Ca(BF_4_)_2_‐based hybrid electrolyte was employed in combination with a small‐molecule, covalently bonded sulfur cathode [14]. The Ca−S batteries with this LiPF_6_/Ca(BF_4_)_2_‐based hybrid electrolyte delivered a cell voltage of ∼2.0 V at a current density of 50 mA g^−1^ and an initial discharge capacity of 824.6 mAh g^−1^ at 100 mA g^−1^ with a retention of 47.9% after 145 cycles. These examples clearly demonstrate the potential of hybrid electrolyte systems for Ca−S batteries. However, no post‐Li‐ion hybrid electrolyte systems have been reported to date, and the characteristic discharge plateau of Ca−S batteries has yet to be achieved. Moreover, in most hybrid electrolytes, the Li^+^ concentration exceeds that of Ca^2+^, and the irreversible consumption of Li^+^ mediators through Li_2_S formation limits cycling stability and contributes to capacity fading.

Herein, we report for the first time a reversible room‐temperature Ca−S battery enabled by a sodium‐borate‐modified calcium borate electrolyte, representing a promising post‐lithium system with enhanced performance. The Ca−S battery was assembled using a modified electrolyte containing a lower concentration of Na^+^ (0.15 M Na[B(hfip)_4_]) than Ca^2+^ (0.3 M Ca[B(hfip)_4_]_2_) in dimethoxyethane (DME), termed as Na‐CaBhfip/DME electrolyte. This electrolyte exhibits high oxidative stability (>4.0 V vs. Ca/Ca^2+^), excellent compatibility with Ca metal, and a low overpotential (<0.1 V) for Ca stripping/plating. The *post mortem* scanning electron microscopy (SEM) coupled with energy‐dispersive x‐ray spectroscopy (EDS) analysis reveals that the Na[B(hfip)_4_] additive induces the formation of a hybrid Na/Ca layer, which effectively mitigates the passivation of the Ca anode. Ca−S coin cells employing this modified electrolyte together with a S/Ketjenblack/Polyaniline composite cathode and Ca metal anode deliver both higher discharge capacity and significantly improved cycling stability. Ex situ x‐ray photoelectron spectroscopy (XPS) analysis confirms the reversible sulfur conversion from S_8_ to Ca polysulfides and finally to CaS, accompanied by the formation of Na polysulfides and Na_2_S, which further activates the sulfur cathode. Moreover, the modified electrolyte enables stable cycling of a Ca−S pouch cell, demonstrating its strong potential for practical Ca−S battery applications.

## 2 Results and Discussion

**FIGURE 1:**
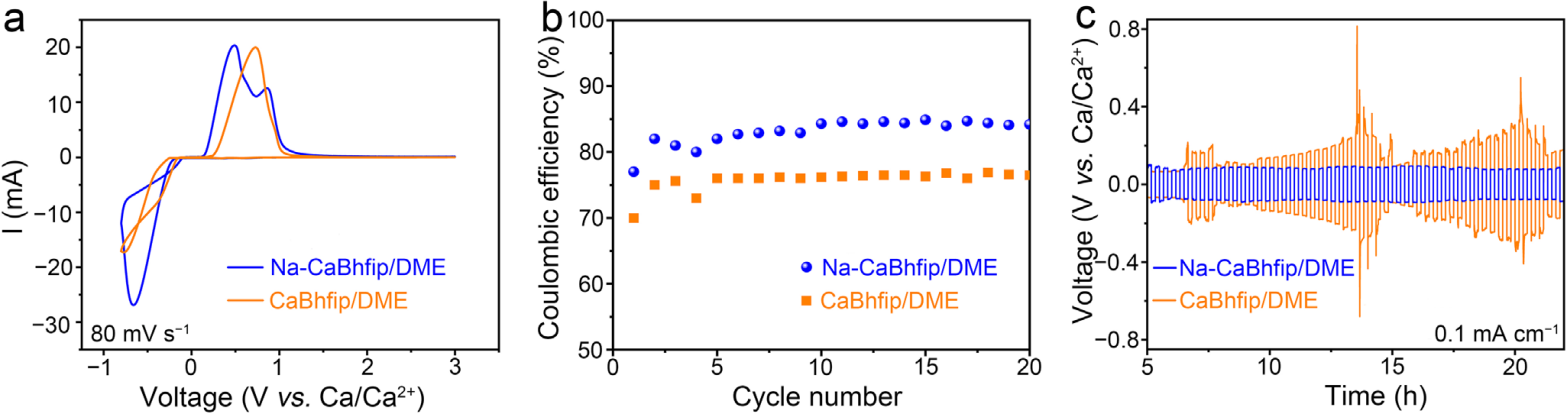
(a) CV curves of the Ca plating/stripping in different electrolytes at a scan rate of 80 mV s^−1^ after conditioning cycles. (b) Coulombic efficiencies calculated from CV curves. (c) Galvanostatic cycling performance of different electrolytes in symmetric Ca||Ca coin cells at a current density of 0.1 mA cm^−2^.

The Ca[B(hfip)_4_]_2_·4DME and Na[B(hfip)_4_]·3DME salts were synthesized through the one‐step reaction of Ca(BH_4_)_2_·2THF and NaBH_4_ and hexafluoroisopropanol (hfip), respectively, following a procedure slightly modified from our previous reports [11, 15]. The electrolyte solutions were prepared by simply dissolving Ca[B(hfip)_4_]_2_·4DME salt with and without Na[B(hfip)_4_]·3DME in a proper amount of DME, denoted as Na‐CaBhfip/DME and CaBhfip/DME, respectively. The Ca foils used in this study were fabricated in‐house using pressing and rolling procedures to achieve a thickness of ∼ 100 µm [16]. The Ca stripping/plating in electrolytes with and without Na[B(hfip)_4_] was analyzed by cyclic voltammetry (CV) at a scan rate of 80 mV s^−1^, using carbon‐coated aluminum (CCAl) and Ca foils as the working and counter electrodes, respectively. The Na‐CaBhfip/DME electrolyte is capable of efficient Ca plating/stripping in the voltage range of −0.8 V–3.0 V at a scan rate of 80 mV s^−1^ (Figure 1a). With the addition of the Na salt, a pair of peaks with an increased current response (>20 mA cm^−2^) was observed compared to the CaBhfip/DME electrolyte (<20 mA cm^−2^), indicating efficient Ca deposition and a higher ionic conductivity of the Na‐CaBhfip/DME electrolyte. The Coulombic efficiency (CE) of Ca deposition was measured based on the charge of each CV scan. The CE increased after a few cycles, and the Na‐CaBhfip/DME electrolyte exhibited a higher CE of ∼84% than the CaBhfip/DME electrolyte (∼76%) in the continuous cycles (Figure 1b). The low CE of the CaBhfip/DME electrolyte is mainly due to the passivation of Ca metal caused by the decomposition of the electrolyte. The cyclability of the modified electrolyte was investigated in symmetric Ca||Ca cells by galvanostatic cycling at a constant current density of 0.1 mA cm^−2^ (Figure 1c). The Na‐CaBhfip/DME electrolyte demonstrates low overpotential (<0.1 V) for more than 200 h (Figure S1). Furthermore, the modified electrolyte was evaluated at different Na[B(hfip)_4_] concentrations (0.05, 0.15, and 0.30 M Na[B(hfip)_4_]) (Figure S1a). As shown in Figure S2a, the Ca metal cycled in 0.05 M Na[B(hfip)_4_] in 0.3 M Ca[B(hfip)_4_]_2_/DME still shows porous Ca deposition morphology, indicating low concentration of Na ions (0.05 M) is insufficient to prevent the passivation of Ca metal and leads to the same behavior as Ca[B(hfip)_4_]_2_. In contrast, increasing the Na[B(hfip)_4_] concentration to 0.30 M results in the formation of a thick Ca/Na hybrid interphase layer on the Ca surface (Figure S2b). Although Ca/Na hybrid layer formation is promoted, excessive Na^+^ leads to a higher plating/stripping overpotential, and subsequently leads to a short circuit after a few cycles. The results indicate that the optimal composition is 0.15 M Na[B(hfip)_4_] in combination with 0.3 M Ca[B(hfip)_4_]_2_, as this formulation exhibited the lowest overpotential (Figure S1b). The linear sweep voltammetry (LSV) of the both electrolytes using stainless steel as the WE and Ca as the RE and CE at a scan rate of 1 mV s^−1^ exhibits a broad electrochemical window up to 4 V versus Ca/Ca^2+^ (Figure S3), which is sufficient for the operating voltage of the Ca−S battery (<3.5 V). This confirms that the Na‐CaBhfip/DME electrolytes are suitable for Ca−S batteries.

**FIGURE 2:**
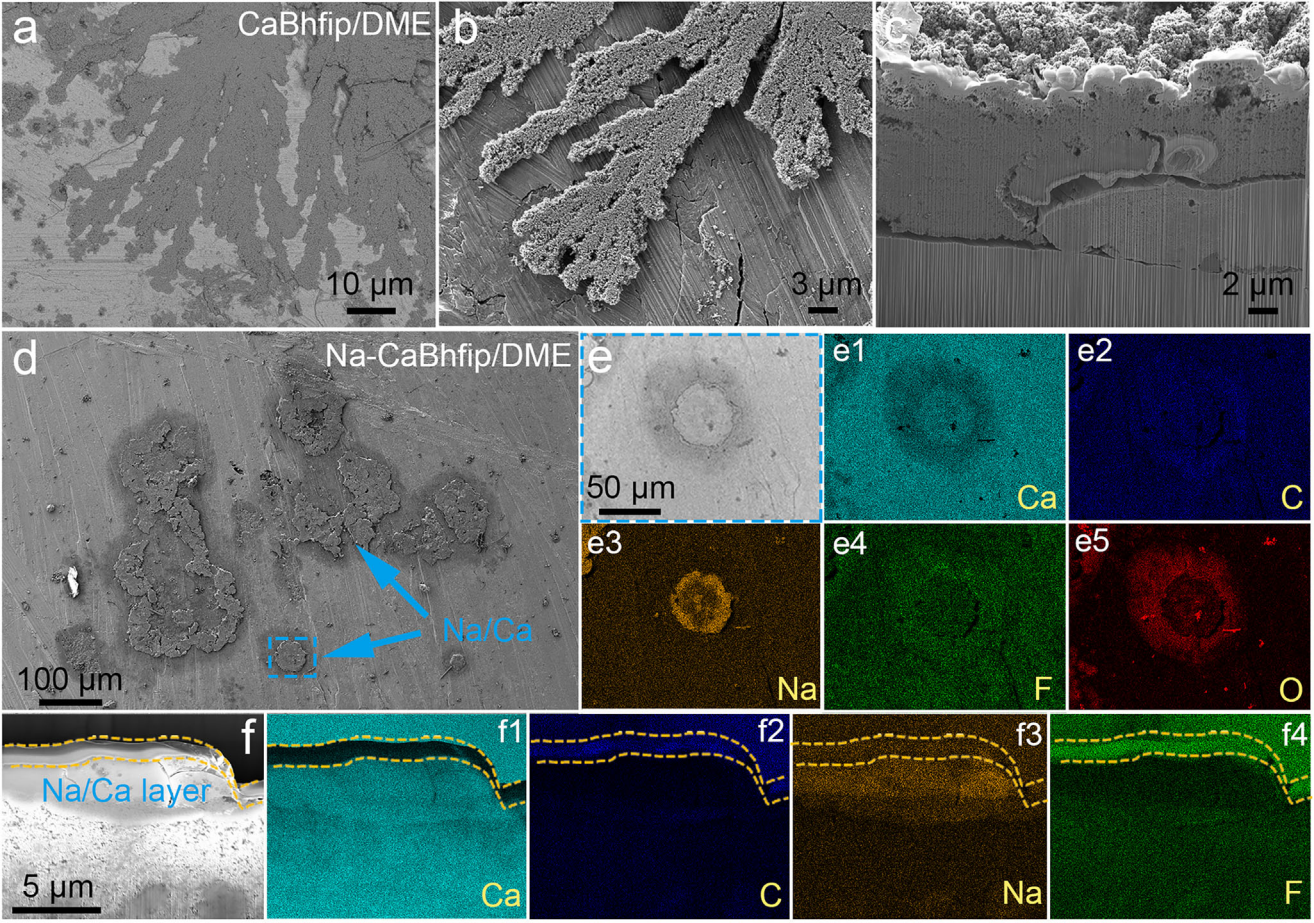
(a, b) SEM images and (c) FIB‐SEM cross‐section image of the plated Ca anode after plating/stripping for 50 cycles in the CaBhfip/DME electrolyte. (d) SEM image of the plated Ca anode after plating/stripping for 50 cycles in the Na‐CaBhfip/DME electrolyte. (e) Enlarged SEM image of an individual particle and the corresponding Ca, C, Na, F, O EDS element mapping images, respectively (e1–e5). (f) FIB‐SEM cross‐section images of the enlarged particle with the corresponding Ca, C, Na, F EDS element mapping images, respectively (f1–f4). The carbon protection layer for the FIB cross sectioning is marked in orange dashed lines.

To further elucidate the impact of the sodium additive on the Ca metal anode, *post mortem* analysis was performed. After disassembling the cells, a black layer was observed on the surface of the Ca anode utilizing CaBhfip/DME as electrolyte, whereas the Na‐CaBhfip/DME electrolyte preserved a shiny silver surface appearance (Figure S4). Morphological and elemental characteristics of the Ca deposits after cycling in the Na‐CaBhfip/DME electrolyte were examined by SEM. Previous reports have shown that native passivation films on the Ca anode in CaBhfip/DME electrolyte include an organic‐rich surface (O−C = O, C−F, C−C species) and an underlying inorganic layer containing CaO, CaH_2_, CaCO_3_, and CaF_2_ [17]. However, the continuous growth of this passivation film during cycling significantly increases charge‐transfer resistance and reduces CE, eventually leading to cell failure. The top‐view SEM images of the Ca anode after 50 cycles in a Ca|CaBhfip/DME|Ca symmetric cell reveal a highly porous deposition morphology with sizes on the order of tens of micrometers (Figure 2a,b). Cross‐sectional focused ion beam−SEM (FIB−SEM) further confirms the presence of a porous structure within the deposited Ca layer (Figure 2c). In several regions, a distinct boundary between the deposited Ca and the pristine Ca anode can be observed, indicating that the plated Ca is loosely stacked on the underlying Ca anode with poor electronic contact. EDS elemental mapping of the deposited Ca shows a heterogeneous distribution of Ca, Na, F, and C (Figure S5), suggesting that Ca deposition occurs in a porous framework accompanied by the formation of a passivation layer. During the stripping process, Ca^2+^ predominantly originates from the electrochemically active Ca metal in the upper layer (Figure S6). However, a fraction of the deposited Ca cannot be reversibly stripped, leading to the formation of so‐called “dead Ca.” The progressive accumulation of dead Ca, together with continuous corrosion of the Ca metal during cycling, ultimately results in electrode failure, which is consistent with previous reports using the same CaBhfip/DME electrolyte [17]. In contrast, with the addition of Na[B(hfip)_4_], the top‐view SEM image of the plated Ca anode after 50 cycles in a Ca||Ca symmetric cell shows a surface covered with irregular particulates ranging from 10 to 100 µm (Figure 2d). An enlarged view of a representative particle (Figure 2e) reveals its dense morphology, and the corresponding EDS elemental maps (Figure 2e1−b5) confirm a distinct Na‐rich region accompanied by fluorinated and oxygenated components, indicative of a hybrid Ca/Na layer induced by Na[B(hfip)_4_] incorporation. Cross‐sectional FIB−SEM imaging of the particle further reveals a dense morphology (Figure 2f). The outer layer (∼1 µm), rich in carbon, is the carbon coating layer to protect the sample during FIB cut. Beneath this, a ∼5 µm layer enriched in both Na and Ca suggests the formation of a hybrid Na/Ca layer, which is thermodynamically plausible given the similar deposition potential of Na^+^ (−2.71 V vs. SHE) and Ca^2+^ (−2.87 V vs. SHE). The Na and Ca atomic ratios were determined to be 5.67 and 52.2 at.% from the surface SEM‐EDS image (Figure 2e), and 3.17 and 83.4 at.% from the cross‐sectional SEM–EDS image (considering only the layer beneath the coating, Figure 2f). These results indicate that Na is relatively enriched at the surface region of the hybrid layer. Together, the symmetric‐cell behavior and FIB‐SEM/EDS analysis demonstrate that Na[B(hfip)_4_] promotes the formation of a protection structure consisting of a hybrid Na/Ca layer. This composite layer effectively suppresses Ca surface passivation and enables stable Ca plating/stripping.

**FIGURE 3:**
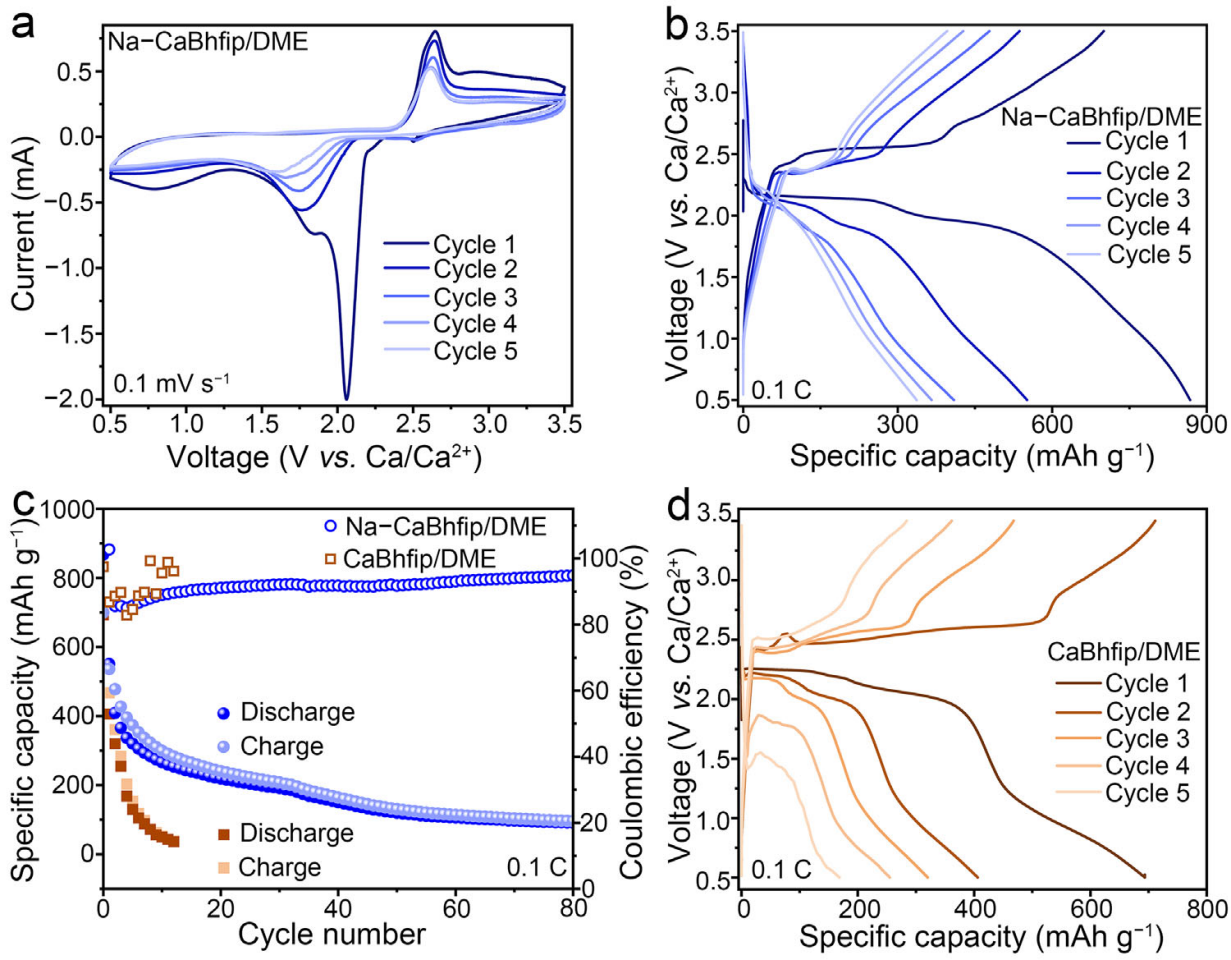
(a) CV curves at 0.1 mV s^−1^. (b) GCD profiles of the Ca||S/PANI coin cell with the Na‐CaBhfip/DME electrolyte at 0.1C. (c) Cycling performance of the Ca||S/PANI cell with the Na‐CaBhfip/DME and CaBhfip/DME electrolyte at 0.1C. (d) GCD profiles of the Ca||S/PANI coin cell with the CaBhfip/DME electrolyte at 0.1C.

To evaluate the impact of the Na‐CaBhfip/DME electrolyte on the redox reaction of the sulfur cathode, Ca−S coin cells using the S/Ketjenblack/Polyaniline composite (S/PANI) as the cathode and Ca foil as the anode were assembled. The S/PANI cathode was prepared by ball‐milling S, PANI, and KB without any additional thermal treatment. The Fourier‐transform‐infrared (FT−IR) spectrum of commercial PANI (Figure S7) shows characteristic bands at 1162 and 823 cm^−1^ corresponding to the benzenoid amine stretch and ring breathing vibration, respectively [18]. These characteristic PANI features remain clearly visible in the S/PANI composite, albeit with slightly reduced intensity, confirming that the polymer backbone is retained and not degraded during the grinding process. Figure 3a shows the CV profiles of the Ca||S/PANI coin cell using the Na‐CaBhfip/DME electrolyte in a voltage window of 0.5−3.5 V (vs. Ca/Ca^2+^) with a scan rate of 0.1 mV s^−1^. During the first scan, two distinct reduction peaks appeared at ∼2.1 and ∼1.8 V, together with a broad signal centered at approximately 0.8 V. The high‐voltage peaks (>2.0 V) can be correlated to the reduction of elemental sulfur to high‐order Ca polysulfides (CaS*_x_*, 4 ≤ x ≤ 8), whereas the low‐voltage features correspond to the stepwise conversion of high‐order Ca polysulfides to lower‐order species (CaS*_x_*, 2 ≤ x< 4), accompanied by the formation of CaS_2_ and CaS [9]. A weak cathodic peak is observed at approximately 2.5 V (vs. Ca/Ca^2+^) during the initial reduction scan. This peak originates from the *p*‐type redox behavior of PANI, as evidenced in Figure S8a. The PANI electrode delivers a minor specific capacity of approximately 60 mAh g^−1^ during the first discharge, which rapidly decreases to about 10 mAh g^−1^ after 30 cycles (Figure S8b). In the subsequent anodic scan, a sharp oxidation peak appears at ∼2.7 V and a broad feature at ∼2.9 V, which can be assigned to the conversion of CaS and CaS_2_ back to higher‐order Ca polysulfides (CaS*_x_*, x > 2) and eventually to elemental sulfur. After initial activation, the CV curves show clearly defined redox pairs from the second cycle onwards, indicating good reversibility of the sulfur redox chemistry in the Na‐CaBhfip/DME electrolyte. Nevertheless, the current densities are significantly lower than those observed in the first scan, which can be attributed to the loss of active sulfur species through the “shuttle effect” of soluble Ca polysulfides, as commonly reported for other metal−sulfur systems [19, 20]. In Figure 3b, the galvanostatic charge/discharge (GCD) curve of the Ca|Na‐CaBhfip/DME|S/PANI coin cell at 0.1C (1C = 1672 mA g^−1^) exhibits two distinct discharge plateaus at ∼2.2 and ∼1.9 V, followed by a sloping region below ∼1.7 V, indicative of multistep sulfur redox reactions. During charging, a flat plateau at ∼2.6 V and a subsequent sloping region above ∼2.7 V are observed. These features are consistent with the CV results of the coin cell.

The cycling performance of the Ca−S coin cells with and without Na additive at 0.1C is presented in Figure 3c. Capacities of 867.7 and 693.5 mAh g^−1^ were observed in the first discharge process of the Ca−S coin cells with the Na‐CaBhfip/DME and CaBhfip/DME electrolyte, respectively. These values are in line with other reports on sulfur composite cathodes in Ca−S coin cells [9]. For the cells with the CaBhfip/DME electrolyte, the discharge capacity rapidly decays upon cycling, and the two characteristic plateaus progressively fade (Figure 3d). This behavior is in agreement with the literature and can be ascribed to the strong activation process coupled with Ca stripping at the Ca metal anode [21]. With the addition of Na[B(hfip)_4_] electrolyte, the stability and capacity are improved dramatically. The Ca|Na‐CaBhfip/DME|S/PANI coin cell shows a discharge capacity of 92 mAh g^−1^ after 80 cycles at 0.1C and the cell voltage of 2.2 V is maintained even after 80 cycles (Figure S9). Although PANI has some redox activity with Na, the PANI cathode delivered only a discharge capacity of 12.1 mAh g^−1^ after 50 cycles at a current density of 300 mA g^−1^ in 0.15 M Na[B(hfip)_4_] electrolyte and a voltage window of 0.5–3.5 V versus Ca^2+^/Ca, as shown in Figure S10. Similarly, in CaBhfip/DME electrolyte, a discharge capacity of 14.5 mAh g^−1^ was obtained under the same conditions (As shown in Figure S8). Considering that the PANI content in the S/PANI composite cathode is approximately 20 wt%, even though only 10% of the initial capacity is retained after 80 cycles for the S/PANI cathode, the contribution from PANI in the S/PANI cathode would account for less than 10% of the final capacity. This indicates that the overall capacity is predominantly derived from the redox reactions of sulfur, rather than from PANI.

The electrochemical performance of Ca|Na‐CaBhfip/DME|S/PANI coin cell at 0.1C with a higher sulfur loading around 3 mg cm^−2^ and electrolyte to sulfur ratio of ∼29 µL mg^−1^ was measured, as shown in Figure S11. The coin cell delivers an initial discharge capacity of 409.4 mAh g^−1^ and maintains reversible cycling over 50 cycles at 0.1C, with a CE stabilizing at around 90%−95% after the initial activation stage. These results demonstrate that the Na‐CaBhfip/DME electrolyte remains electrochemically active and can support sulfur conversion under high sulfur loading. As summarized in Table S1, our work introduced a lower concentration of Na[B(hfip)_4_] relative (0.15 M) to Ca[B(hfip)_4_]_2_ (0.3 M), the Ca−S coin cell with the Na‐CaBhfip/DME electrolytes exhibited a discharge voltage of ∼2.2 V (close to the thermodynamic value) and good sulfur conversion reversibility compared to most previously reported systems, in which the concentrations of Li or Na salts were typically higher than that of the Ca salt [9, 10, 12, 14, 21, 22]. These results clearly demonstrate the impact of the Na[B(hfip)_4_]‐modified Ca[B(hfip)_4_]_2_ electrolyte for Ca−S batteries.

**FIGURE 4:**
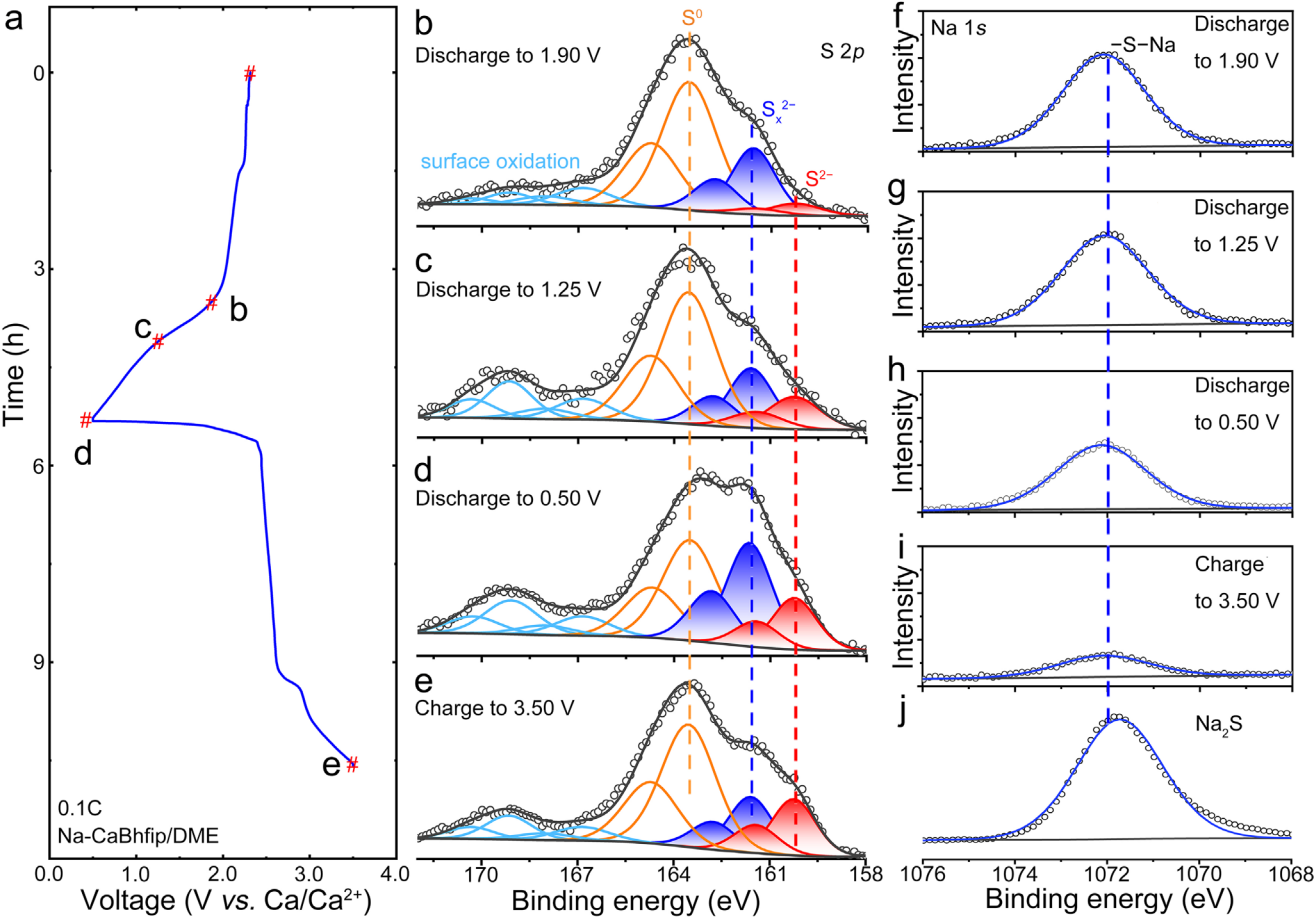
(a) Voltage profiles of galvanostatic cycling of a Ca|Na‐CaBhfip/DME|S/PANI coin cell at a current density of 0.1C in the voltage window of 0.5−3.5 V (vs. Ca/Ca^2+^). High‐resolution S 2*p* XPS spectra of the cathodes at discharge to (b) 1.90, (c) 1.25, (d) 0.50, and (e) charge to 3.5 V. High‐resolution Na 1*s* XPS of the cathodes at discharge to (f) 1.90, (g) 1.25, (h) 0.50, and (i) charge to 3.5 V, and (j) commercial Na_2_S sample.

To gain deeper insight into the sulfur conversion mechanism of the Ca−S battery with the Na‐CaBhfip/DME electrolyte, the evolution of the oxidation state of sulfur in the cathode during galvanostatic cycling (0.1C) at different charge/discharge states (Figure 4a) was analyzed by XPS. The S 2*p* detail spectra of the discharge and charge products provide the crucial information, as shown in Figures 4b–e for the discharged sulfur cathode of the Ca|Na‐CaBhfip/DME|S/PANI coin cell. Elemental sulfur (S_8_) typically exhibits a S 2*p* peak doublet at 164.0 eV (2*p*_3/2_) and 165.2 eV (2*p*_1/2_), as shown in the high resolution S 2*p* XPS spectrum of the pristine electrode (Figure S12) [14]. At a discharge voltage of 1.9 V (Figure 4b), the S 2*p* spectrum could be mainly deconvoluted into three doublets. The smaller and relatively broad feature at higher binding energy (≈168−169 eV) can be attributed to oxidized S species and was also observed in other studies [23]. The doublet for S_8_ is still detected, whereas the feature at 160.3/161.5 eV can be assigned to metal sulfide S^2−^ and the doublet at 161.6/162.8 eV to metal disulfide S_2_^2−^ and the terminal S atoms of the metal polysulfides S*_x_* ^2−^ (2< x < 8), respectively. Upon further discharge to 1.25 V (Figure 4c) and 0.5 V (Figure 4d), the signal intensity of elemental sulfur decreases successively while that of metal disulfide/polysulfides increases, reflecting the effective conversion of sulfur to metal polysulfides and finally to metal sulfides. Upon recharging to 3.5 V (Figure 4e), the peaks for S_8_ are restored to a large extent, evidencing the reversible conversion of metal sulfide back to sulfur. The sulfur (S^0^): polysulfide (S*_x_* ^2−^): sulfide (S^2−^) ratios at the pristine state, discharged to 1.9, discharged to 1.25, discharged to 0.5, and charged to 3.5 V were determined to be 1:0:0, 0.627:0.228:0.145, 0.440:0.379:0.181, and 0.667:0.276:0.057, respectively. These results clearly demonstrate the reversible conversion of sulfur to polysulfides and subsequently to sulfides during cycling. The Ca 2*p* detail spectra (Figure S13) show, as expected, no detectable Ca signal for the pristine electrode. Upon discharge and charge, the Ca 2*p* peak doublet appears at 347.9 and 351.4 eV [24], consistent with the formation of Ca polysulfides and CaS_2_/CaS. In the Na 1*s* spectrum of the pristine electrode (Figure S14), a broad peak was observed at 1073.3 eV, attributed to the –C–O–Na interaction from the sodium carboxymethyl cellulose binder [25]. During charge/discharge, a new Na 1*s* peak is found at a lower binding energy of 1072.1 eV (Figure 4f−i), which can be ascribed to Na_2_S (Figure 4j) [26]. Quantitative peak fitting of the S *2p*, Ca *2p*, and Na *1s* XPS spectra collected at different charge/discharge states is summarized in Table S2. The XPS spectra were normalized using the carbon signal as a reference, as shown in Table S3, assuming that the carbon content remains unchanged throughout cycling. As summarized in Tables S2 and S3, the contents of both Ca and Na species increase during discharge and decrease upon charging, following the evolution of sulfur species. These results provide direct evidence that both Ca^2+^ and Na^+^ participate in the sulfur redox reactions and undergo reversible conversion reactions during cycling. EDS measurements (Figure S15) show no detectable Ca signal and only a weak Na signal in the pristine cathode, whereas both Ca and Na appear with high intensity in the fully discharged state. Upon subsequent charging to 3.5 V, the intensities of Ca and Na decrease again. The atomic percentages of Ca and Na increased from ∼0.00 and 0.34 at.% in the pristine state to 11.99 and 1.02 at.% in the fully discharged state, respectively, and then decreased to 6.55 and 0.31 at.% after recharging, as shown in Figure S16. These results suggest that the discharge–charge process involves reversible sodiation/desodiation of sulfur accompanied by calciation/decalciation reactions. Overall, the XPS and EDS results confirm the reversible multistep redox process between S_8_, polysulfides, and CaS_2_/CaS in the Ca−S system with the Na‐CaBhfip/DME electrolyte. Additionally, Na polysulfides and Na_2_S were generated from the Na[B(hfip)_4_] additive during cycling. The enhanced sulfur utilization and cycling performance of the Ca–S cells with the additional Na salt, as shown in Figure 3, indicates that the Na additive can promote the reversibility of the sulfur conversion reaction in Ca‐based batteries.

**FIGURE 5:**
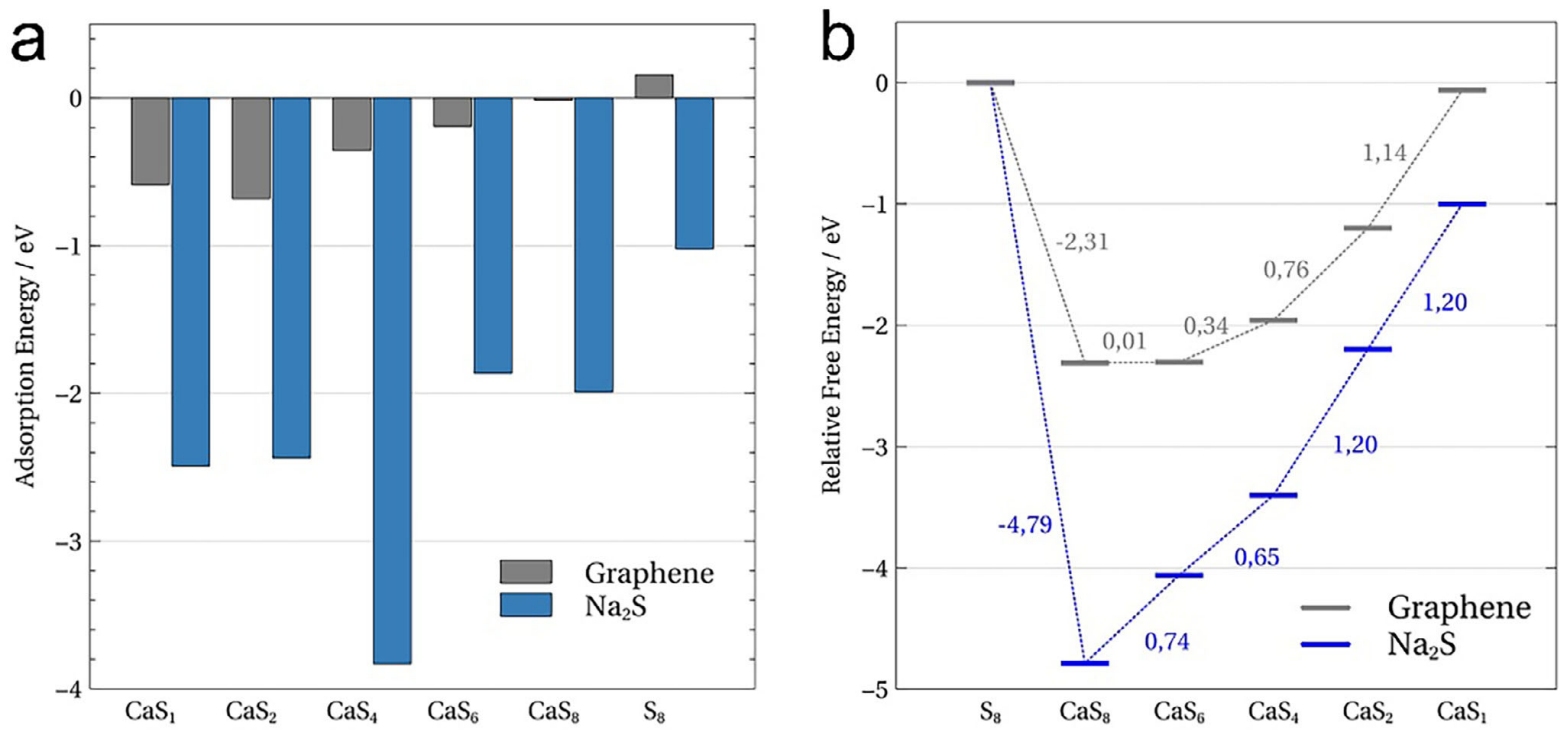
(a) Binding energy (in eV) of the S_8_ and representative Ca polysulfide species (CaS, CaS_2_, CaS_4_, CaS_6_, and CaS_8_) adsorbed on graphene and Na_2_S surface. (b) DFT calculations of the relative free energy diagram during the discharge process from S_8_ to CaS on the surface of Na_2_S and graphene.

The impact of Na^+^ on the Ca−S conversion reaction was further studied by density functional theory (DFT) calculations. Similar to the report verifying the enhanced Ca−S conversion through the involvement of Na^+^ in the sulfur redox reactions [10], we also found that Na^+^ participates in the sulfur conversion process in our study. The presence of the signals at 160.3/161.5 eV in the S 2*p* and 1072.1 eV in Na 1*s* XPS spectra of the Ca−S batteries discharged to 1.9 V in the Na‐CaBhfip/DME electrolyte indicates the formation of Na_2_S. To simplify the theoretical understanding of how the formed Na polysulfide/sulfide influences Ca−S conversion, Na_2_S was selected as a representative species in the DFT calculations to investigate its effect on the sulfur conversion reactions with Ca^2+^. We first calculated the adsorption energies of S_8_ and representative Ca polysulfide species (CaS, CaS_2_, CaS_4_, CaS_6_, and CaS_8_) on graphene and Na_2_S surfaces. Figure S17 and Figure S18 present the optimized adsorption configurations of S_8_ and the representative Ca polysulfides on the surface of Na_2_S and graphene, respectively. The corresponding binding energies are summarized in Figure 5a. The results show that Na_2_S exhibits stronger binding toward all Ca polysulfide species compared with graphene. Furthermore, the relative free‐energy diagrams for the sulfur conversion reactions on graphene and Na_2_S surfaces were calculated, as shown in Figure 5b. In general, the formation of all Ca polysulfide intermediates is thermodynamically more favorable on the Na_2_S surface than on graphene, as evidenced by the lower relative free‐energy values. The DFT results indicate that the Na polysulfide/sulfide species formed due to the involvement of the Na^+^ in the sulfur redox reactions can enhance the adsorption of Ca polysulfides and promote the Ca–sulfur conversion. These findings are consistent with the results of Ca–S coin cells, where the presence of Na^+^ in the electrolyte improves the reversibility of the sulfur cathode.

**FIGURE 6:**
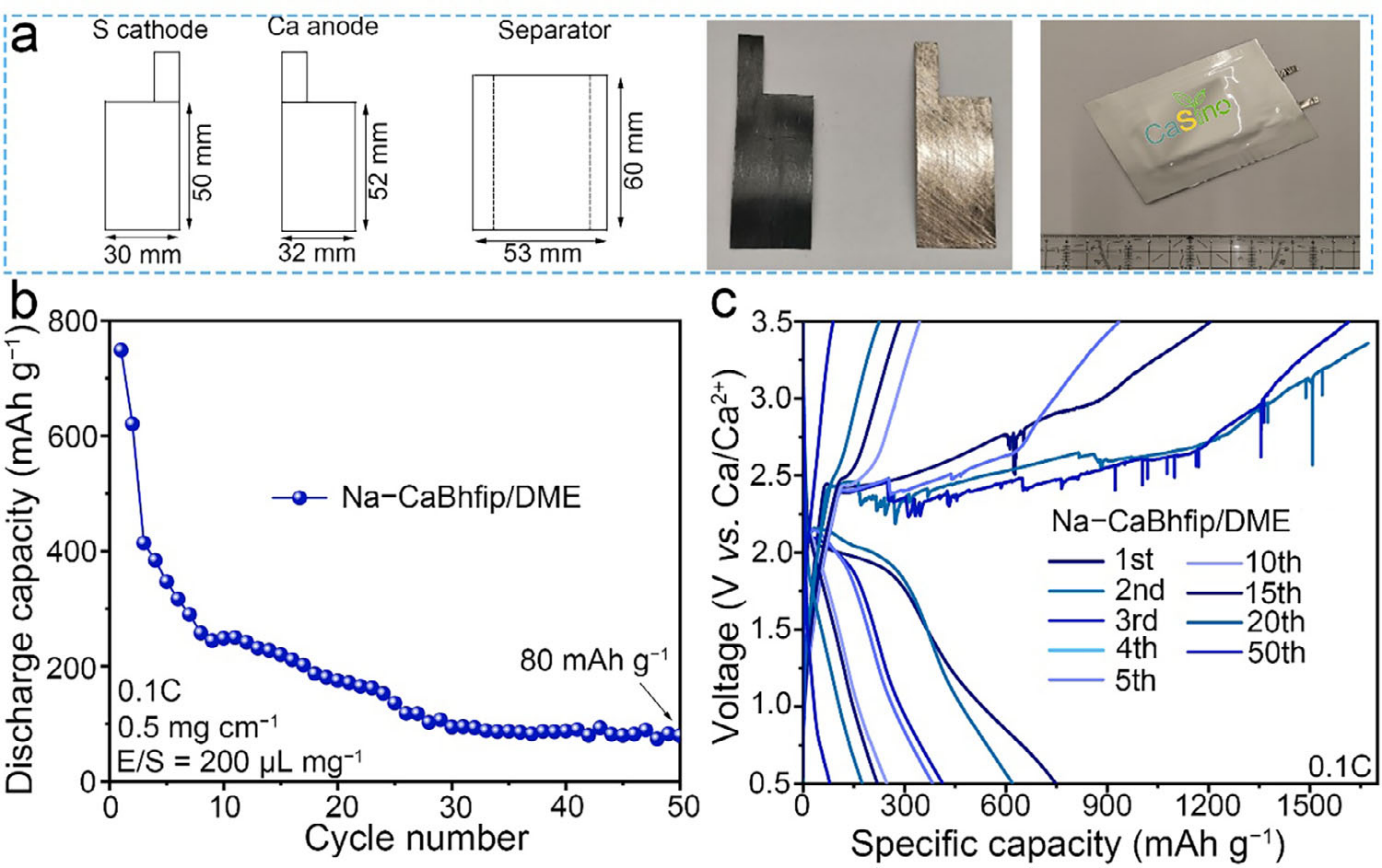
(a) Schematic and photographs of the sulfur cathode, calcium anode, separator, and the assembled Ca−S pouch cell. Cycling performance (b) and GCD profiles (c) of the Ca−S pouch cell with the Na‐CaBhfip/DME electrolyte.

To further validate the integration of the Na‐CaBhfip/DME electrolyte in a practical cell configuration, lab‐scale pouch cells were successfully assembled in a format of 53 × 60 mm^2^ as shown in Figure 6a. In the pouch cells employing the CaBhfip/DME electrolyte, an S/PANI cathode with a sulfur loading of 1 mg cm^−2^ and 0.75 mL of CaBhfip/DME electrolyte was used. The specific capacity value was calculated based on the mass of sulfur in the composite. As shown in Figure S19a, the pouch cell employing the CaBhfip/DME electrolyte shows an initial specific discharge capacity of approximately 750 mAh g^−1^ at 0.1C, which decreases to around 150 mAh g^−1^ by the second cycle and further declines to nearly 0 mAh g^−1^ after 20 cycles. GCD profiles shows the pouch cell with pure CaBhfip/DME electrolyte suffers from severe capacity fading, low sulfur utilization (<100 mAh g^−1^), and the sloping voltage profiles with large polarization (Figure S19b). In the pouch cells employing the Na‐CaBhfip/DME electrolyte, the sulfur mass loading on the cathode was 0.5 mg cm^−2^, with 1.5 mL of electrolyte used to evaluate the feasibility of the Na‐CaBhfip/DME electrolyte in Ca−S pouch cell. Figure 6b displays the cycling performance of the Ca−S pouch cell at 0.1C, the pouch cell with Na‐CaBhfip/DME electrolyte delivers a similar initial capacity of ∼750 mAh g^−1^, but maintains a reversible capacity of ∼80 mA h g^−1^ after 50 cycles, demonstrating the beneficial effect of Na‐CaBhfip/DME electrolyte on cycle life and sulfur utilization in this lab‐scale pouch cell demonstrator. Figure 6c presents the GCD curves of the Ca−S pouch cell with Na‐CaBhfip/DME at 0.1C. Stable and well‐defined voltage plateaus are observed during repeated cycles, indicating reversible sulfur redox processes. The CE of the pouch cell with Na‐CaBhfip/DME electrolyte is shown in Figure S20, where the CE during the initial 10 cycles is very high and remains above 100% throughout the subsequent cycling. This behavior suggests a more pronounced polysulfide shuttle effect in the pouch‐cell configuration compared with coin cells, which is likely associated with the increased sulfur content in the scaled‐up cell configuration. These results indicate that, although the Na‐CaBhfip/DME can enable the operation of Ca−S pouch cells, suppressing polysulfide shuttling remains a key challenge for further performance improvement. To the best of our knowledge, there are few reports about the performance of Ca−S batteries or Ca batteries in a pouch cell configuration. The results further confirm that the Na‐CaBhfip/DME electrolyte can be used on a large‐scale application for developing Ca−S systems.

**FIGURE 7:**
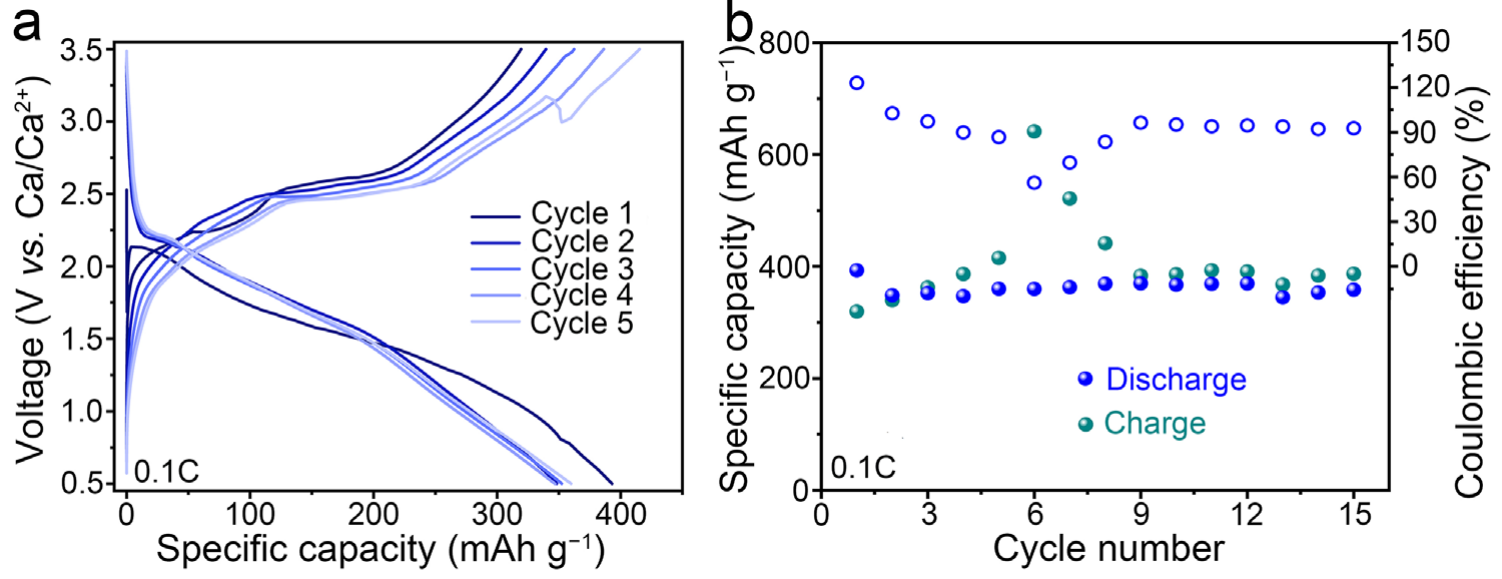
(a) GCD profiles and (b) cycling performance of the Ca||SPANI coin cell with Na‐CaBhfip/DME electrolyte at 0.1C.

Although Ca−S batteries with the Na‐CaBhfip/DME electrolyte achieved high initial specific capacity, cycling stability remains unsatisfactory due to the formation of soluble Ca polysulfides freely shuttling from cathode to anode, leading to the loss of active materials and the passivation of the Ca anode. To further improve the cycling stability of Ca−S batteries with Na‐CaBhfip/DME electrolyte, a heated S/PANI cathode (SPANI) with covalently bonded sulfur species on the polymer backbone was prepared. As previously reported for Li−S systems, the sulfurization process of PANI introduces abundant polar S‐containing functional groups (e.g., C−S, S−S species) on the PANI backbone, which enables robust Li polysulfides trapping via strong covalent bonds [27]. The SPANI electrode was synthesized through in situ sulfurization of PANI by thermal treatment at 300°C under an Ar atmosphere using the S/PANI composite as the precursor. As shown in Figure S21, the FT−IR spectrum of the SPANI composite exhibits pronounced differences compared to pristine PANI. The characteristic C = C stretching vibration of the benzenoid rings at 1497 cm^−1^ shifts to a lower wavenumber, likely due to the substitution of hydrogen atoms on the aromatic rings by sulfur atoms. Similarly, the C−N stretching bands between 1400 and 1200 cm^−1^ also move toward lower frequencies. Meanwhile, the C−H vibration near 1172 cm^−1^ markedly weakens, further supporting the replacement of aromatic hydrogen by sulfur. Additionally, a new absorption band emerges around 926 cm^−1^. Since elemental sulfur is IR‐inactive in the 900−2000 cm^−1^ range, this new feature is attributed to the formation of aromatic (Ar)−S bonds [28]. The electrochemical performance of the SPANI electrode was investigated using the Na‐CaBhfip/DME electrolyte and Ca metal anode. The discharge/charge curves of the first five cycles show almost complete overlap with less capacity loss, as presented in Figure 7a. The initial discharge capacity of this cell is lower than that of the cell with the S/PANI electrode because only a fraction of sulfur atoms in the polymer framework are electrochemically active, while the rest are covalently bound and cannot fully participate in reversible redox reactions [29]. At a current density of 0.1C, the discharge capacity of the first cycle was 393.1 mA h g^−1^, and it remained at 349.2 mA h g^−1^ (88.8% of initial capacity) after 15 cycles with high CE (Figure 7b). In contrast, the cell using the CaBhfip/DME electrolyte still exhibits rapid capacity decay during the initial 10 cycles (Figure S22). The discharge capacity of the Ca||SPANI coin cell with CaBhfip/DME electrolyte at 0.1C decreases dramatically from approximately 500 mAh g^−1^ in the first cycle to below 100 mAh g^−1^ within 10 cycles, accompanied by severe polarization growth. These results clearly indicate that suppressing polysulfide shuttling alone is insufficient to maintain stable Ca−S battery cycling due to the strong passivation of the Ca anode. In contrast, the introduction of the Na‐containing additive effectively stabilizes the Ca anode/electrolyte interface and enables more stable cycling of Ca||SPANI cell with minor capacity decay for 15 cycles. Overall, combining the advantages of Na[B(hfip)_4_]‐modified electrolyte and SPANI cathode, the Ca−S coin cell demonstrates good electrochemical properties and cycling stability.

## 3 Conclusion

In conclusion, we have developed an efficient borate‐based electrolyte, Ca[B(hfip)_4_]_2_ modified with Na[B(hfip)_4_], for improved rechargeable Ca−S batteries. The Na[B(hfip)_4_] additive plays a crucial dual role in enhancing the redox conversion of the sulfur cathode and stabilizing the Ca metal anode. Benefiting from this synergistic effect, a Ca−S coin cell with a S/Ketjenblack/polyaniline cathode and Ca metal anode delivers a high initial discharge capacity of 867.7 mAh g^−1^ at 0.1C and maintains a stable discharge voltage of around 2.2 V for 80 cycles. Moreover, the modified electrolyte enables stable operation of a Ca−S pouch cell for 50 cycles, achieving a discharge capacity of 80 mAh g^−1^ at 0.1C, marking the first successful demonstration of a rechargeable Ca−S pouch cell. In addition, a sulfurized polyaniline composite was designed to further suppress the polysulfide shuttle, contributing to improved cycling stability. This electrolyte engineering strategy, which simultaneously enhances sulfur cathode kinetics and mitigates Ca anode passivation, provides new insights into the rational design of next‐generation electrolytes for Ca‐based batteries.

## Conflicts of Interest

The authors declare no conflict of interest.

## Supporting information




**Supporting File**: smll74655‐sup‐0001‐SuppMat.docx.

## Data Availability

The data that support the findings of this study are openly available at Zenodo https://doi.org/10.5281/zenodo.20496690.

## References

[1] X. Liu , X. Dong , H. Adenusi , Y. Wu , and S. Passerini , “Co‐Solvent Strategy for Rechargeable Post‐Lithium Metal Batteries,” Nature Reviews Chemistry 9 (2025): 415–426, 10.1038/s41570-025-00714-6. 40295892

[2] B. Esser , H. Ehrenberg , M. Fichtner , A. Groß , and J. Janek , “Post‐Lithium Storage—Shaping the Future,” Advanced Energy Materials 15 (2024): 2402824, 10.1002/aenm.202402824.

[3] A. Ponrouch , C. Frontera , F. Barde , and M. R. Palacin , “Towards a Calcium‐Based Rechargeable Battery,” Nature Materials 15 (2016): 169–172, 10.1038/nmat4462. 26501412

[4] M. Wang , C. Jiang , S. Zhang , X. Song , Y. Tang , and H. M. Cheng , “Reversible Calcium Alloying Enables a Practical Room‐Temperature Rechargeable Calcium‐Ion Battery With a High Discharge Voltage,” Nature Chemistry 10 (2018): 667–672, 10.1038/s41557-018-0045-4. 29686378

[5] Z. Zhao‐Karger , Y. Xiu , Z. Li , A. Reupert , T. Smok , and M. Fichtner , “Calcium‐Tin Alloys as Anodes for Rechargeable Non‐Aqueous Calcium‐Ion Batteries at Room Temperature,” Nature Communications 13 (2022): 3849, 10.1038/s41467-022-31261-z. PMC9253317 35788588

[6] H. Lin , Z. Zhan , H. Zeng , et al., “Ultrastable Calcium Metal Anodes Enabled by a Strongly Coordinated Electrolyte Derived Bilayer Solid Electrolyte Interphase,” Advanced Materials (2025): 10711, 10.1002/adma.202510711. 41055306

[7] Z. Li , S. Cui , J. Hacker , et al., “Calcium Chemistry as a New Member of Post‐Lithium Battery Family: What Can We Learn From Sodium and Magnesium Systems,” Angewandte Chemie International Edition 64 (2025): 202415942, 10.1002/anie.202415942. 39679638

[8] C. Ye , H. Li , Y. Chen , et al., “The Role of Electrocatalytic Materials for Developing Post‐Lithium Metal||Sulfur Batteries,” Nature Communications 15 (2024): 4797, 10.1038/s41467-024-49164-6. PMC11535197 38839870

[9] Z. Li , B. P. Vinayan , T. Diemant , R. J. Behm , M. Fichtner , and Z. Zhao‐Karger , “Rechargeable Calcium–Sulfur Batteries Enabled by an Efficient Borate‐Based Electrolyte,” Small 16 (2020): 2001806, 10.1002/smll.202001806. 32812367

[10] D. Zhou , X. Tang , X. Zhang , et al., “Multi‐Ion Strategy Toward Highly Durable Calcium/Sodium–Sulfur Hybrid Battery,” Nano Letters 21 (2021): 3548–3556, 10.1021/acs.nanolett.1c00448. 33851851

[11] Z. Li , O. Fuhr , M. Fichtner , and Z. Zhao‐Karger , “Towards Stable and Efficient Electrolytes for Room‐Temperature Rechargeable Calcium Batteries,” Energy & Environmental Science 12 (2019): 3496–3501, 10.1039/C9EE01699F.

[12] K. A. See , J. A. Gerbec , Y. S. Jun , F. Wudl , G. D. Stucky , and R. Seshadri , “A High Capacity Calcium Primary Cell Based on the Ca–S System,” Advanced Energy Materials 3 (2013): 1056–1061, 10.1002/aenm.201300160.

[13] X. Yu and A. Manthiram , “Toward Reversible Room‐Temperature Calcium‐Ion Batteries,” Chemistry 4 (2018): 1200–1202, 10.1016/j.chempr.2018.05.009.

[14] S. Wu , Z. Jiang , C. Wu , et al., “High‐Content Carbon Layer Confined Small‐Molecule/Covalent Sulfur Cathode Enables Long‐Life Calcium–Sulfur Batteries in Hybrid‐Ion Electrolyte,” Advanced Functional Materials 34 (2024): 2313441, 10.1002/adfm.202313441.

[15] A. Roy , M. Sotoudeh , S. Dinda , et al., “Improving Rechargeable Magnesium Batteries Through Dual Cation Co‐Intercalation Strategy,” Nature Communications 15 (2024): 492, 10.1038/s41467-023-44495-2. PMC10786895 38216573

[16] R. Sibylle , L. Kahnt , C. Kiesl , et al.Batteries & Supercaps 9 (2026): e202500822, 10.1002/batt.202500822.

[17] H. Lin , J. Meng , W. Guo , et al., “Deciphering the Dynamic Interfacial Chemistry of Calcium Metal Anodes,” Energy & Environmental Science 17 (2024): 6548–6558, 10.1039/D4EE01257G.

[18] R. Zou , W. Liu , and F. Ran , “Selenium‐Doped Cathode Materials With Polyaniline Skeleton for Lithium‐Organosulfur Batteries,” Journal of Energy Chemistry 79 (2023): 148–157, 10.1016/j.jechem.2022.12.027.

[19] L. Wang , Z. Li , Z. Meng , et al., “Designing Gel Polymer Electrolyte With Synergetic Properties for Rechargeable Magnesium Batteries,” Energy Storage Materials 48 (2022): 155–163, 10.1016/j.ensm.2022.03.006.

[20] L. Wang , S. Riedel , A. Welle , et al., “Advancing Reversible Magnesium−Sulfur Batteries With a Self‐Standing Gel Polymer Electrolyte,” ACS Applied Energy Materials 7 (2024): 5857–5868, 10.1021/acsaem.4c01049.

[21] A. Scafuri , R. Berthelot , K. Pirnat , et al., “Spectroscopic Insights Into the Electrochemical Mechanism of Rechargeable Calcium/Sulfur Batteries,” Chemistry of Materials 32 (2020): 8266–8275, 10.1021/acs.chemmater.0c02074.

[22] X. Yu , M. J. Boyer , G. S. Hwang , and A. Manthiram , “Toward a Reversible Calcium‐Sulfur Battery With a Lithium‐Ion Mediation Approach,” Advanced Energy Materials 9 (2019): 1803794, 10.1002/aenm.201803794.

[23] T. Gao , S. Hou , F. Wang , et al., “Reversible S 0 /MgS x Redox Chemistry in a MgTFSI 2 /MgCl 2 /DME Electrolyte for Rechargeable Mg/S Batteries,” Angewandte Chemie International Edition 56 (2017): 13526–13530, 10.1002/anie.201708241. 28849616

[24] P. Mondal , B. K. Brahma , D. K. Vali , et al., “Calcium‐Based Metal‐Organic Framework: Detection and Idiosyncratic Removal of Copper by Nano‐Particle Deposition,” Chemistry−A European Journal 30 (2024): e202400587, 10.1002/chem.202400587. 38639718

[25] T. Xu , Z. Yao , W. Jiang , et al., “C–Na–O Electrostatic Interactions Boost the Kinetics of Coal‐Derived Hard Carbon Anodes for High‐Performance Sodium‐Ion Batteries,” Journal of Materials Chemistry A 13 (2025): 29471–29485, 10.1039/D5TA03632A.

[26] P. Wei , Y. Yang , C. J. Liu , and H. Y. Gao , “Single‐Monolayer Na 2 S Film on Metal Surfaces,” Advanced Functional Materials 34 (2024): 2409769, 10.1002/adfm.202409769.

[27] Q. Zhang , Q. Huang , S. M. Hao , et al., “Polymers in Lithium–Sulfur Batteries,” Advanced Science 9 (2022): 2103798, 10.1002/advs.202103798. 34741443 PMC8805586

[28] L. Xiao , Y. Cao , J. Xiao , et al., “A Soft Approach to Encapsulate Sulfur: Polyaniline Nanotubes for Lithium‐Sulfur Batteries With Long Cycle Life,” Advanced Materials 24 (2012): 1176–1181, 10.1002/adma.201103392. 22278978

[29] M. S. Ahmed , S. Lee , M. Agostini , et al., “Multiscale Understanding of Covalently Fixed Sulfur–Polyacrylonitrile Composite as Advanced Cathode for Metal–Sulfur Batteries,” Advanced Science 8 (2021): 2101123, 10.1002/advs.202101123. 34369100 PMC8564465

