# Supplementary material for: An Efficient Sodium Borate‐Based Electrolyte Additive to Activate Sulfur Conversion and Stabilize Calcium Anodes in Calcium−Sulfur Batteries
Source: Small. 2026 Jul 21;22(52):e74655. doi: 10.1002/smll.74655 (PMC13580245; doi:10.1002/smll.74655)
Supplement: Supplementary file 1 [file SMLL-22-e74655-s001.docx]

Supporting Information

**An Efficient Sodium Borate-Based Electrolyte Additive to Activate Sulfur Conversion and Stabilize Calcium Anodes in Calcium−Sulfur Batteries**

*Sebahat Topal,^1^ Ping Feng,^1^* Marco Kögel,^2,4^ Sibylle Riedel,^1^ Joachim Haecker,^3^ Maryam Nojabaee,^3^ Thomas Diemant,^1^ Darius Hübner,^5^ Jannik C. Meyer,^2,4^ Timo Jacob,^1,5,6^ Maximilian Fichtner,^1,7^ Zhirong Zhao-Karger^1,7^**

^1^Helmholtz Institute Ulm (HIU) Electrochemical Energy Storage, Helmholtzstraße 11, 89081 Ulm, Germany

^2^NMI Natural and Medical Sciences Institute at the University of Tübingen, Markwiesenstrasse 55, 72770 Reutlingen, Germany

^3^Deutsches Zentrum für Luft- und Raumfahrt e. V. (DLR) German Aerospace Center, Institute of Engineering Thermodynamics, Electrochemical Energy Technology, Pfaffenwaldring 38-40, 70569 Stuttgart, Germany

^4^Institute of Applied Physics, Eberhard Karls University of Tübingen, Auf der Morgenstelle 10, 72076 Tübingen, Germany

^5^Institute of Electrochemistry, Ulm University, Albert-Einstein-Allee 47, 89081, Ulm, Germany

^6^Karlsruhe Institute of Technology (KIT), P.O. Box 3640, 76021 Karlsruhe, Germany

^7^Institute of Nanotechnology, Karlsruhe Institute of Technology (KIT), Hermann-von-Helmholtz-Platz 1, 76344 Eggenstein-Leopoldshafen, Germany

* Corresponding author. E-mail address: ping.feng@kit.edu (*Ping Feng*), zhirong.zhao-karger@kit.edu (*Zhirong Zhao-Karger*).

**Experimental Section:**

All experiments were carried out either on the bench under Ar (99.9999%) using the standard Schlenk technique in an Ar-filled glove box (MBraun) with H_2_O and O_2_ concentration <0.5 ppm or at ambient atmosphere. The following starting materials were used as purchased: Calcium chunks (99%, Thermo Fisher Scientific), Ca(BH_4_)_2_∙2THF (Merck), NaBH_4_ (Merck), DME (99.5%, extra dry, stabilized, Thermo Fisher Scientific), hexafluoroisopropanol (99%, abcr GmbH), polyaniline (average Mw ~50,000), Ketjenblack (EC-600JD, specific surface area ≈ 1400 m^2^ g^−1^).

***Material preparation*** DME was dried over 3 Å molecular sieves overnight before use. The Ca[B(hfip)_4_]_2_·4DME and Na[B(hfip)_4_]·3DME salts were synthesized according to our previous reports with slight modifications.^[1]^ **Ca[B(hfip)_4_]_2_·4DME**: Under argon atmosphere, Ca(BH_4_)_2_∙2THF (5.0 g, 23.4 mmol, 1 eq.) was suspended in dimethoxyethane (100 mL). Hexafluoroisopropanol (22.0 mL, 208 mmol, 8.9 eq.) was then added dropwise (gas evolution) to give a clear solution. The solution was then left to stir overnight at room temperature (rt). The next day, the solvent was removed under reduced pressure (~ 10^−2^ mbar, rt to 60 °C). In an argon-filled glovebox, the resulting white powder was transferred to a BÜCHI tube and dried for 24 h in a BÜCHI oven (~ 10^−2^ mbar, 60 °C, 5 rpm). The product Ca[B(hfip)_4_]_2_∙4DME (37.9 g, 21.55 mmol, 92%) was obtained as a white crystalline powder. The analytical data were in agreement with those reported in the literature. **Na[B(hfip)_4_]·3DME**: Under argon atmosphere, NaBH_4_ (2.0 g, 52.9 mmol, 1 eq.) was dissolved in dimethoxyethane (100 mL), and the solution was cooled to 0 °C. Then hexafluoroisopropanol (24.0 mL, 227 mmol, 4.3 eq.) was added dropwise (gas evolution). The solution was then allowed to warm to room temperature (rt) and heated under reflux for 1 h. It was then stirred overnight at rt. The next day, the solvent was removed under reduced pressure (~ 10^−2^ mbar, rt to 60 °C). In an argon-filled glove box, the resulting white powder was transferred to a BÜCHI tube and dried overnight in a BÜCHI oven (~ 10^−2^ mbar, 60 °C, 5 rpm). The product (Na[B(hfip)_4_]∙3DME; 45.2 g, 46.5 mmol, 88%) was obtained as a white crystalline, slightly sticky powder. The analytical data are in agreement with those reported in the literature. All electrolyte salts were dissolved in 1,2-dimethoxyethane (DME). To prepare 2 mL of [0.15 M Na[B(hfip)_4_]−0.3 M Ca[B(hfip)_4_]_2_/DME] electrolyte, 1.0558 g Ca[B(hfip)_4_]_2_·4DME and 0.2916 g Na[B(hfip)_4_]·3DME were added to 1.24  mL DME. 2 mL of the single salt electrolyte solution, 0.3 M Ca[B(hfip)_4_]_2_/DME, was prepared by adding 1.0558 g of Ca[B(hfip)_4_]_2_·4DME salts to 1.45 mL DME. All electrolyte solutions were stirred for 1 day for further usage. The S/Ketjenblack/Polyaniline composite (S/PANI) was prepared by a ball milling method. KB (0.2 g), PANI (0.2 g), and sulfur (0.5 g) were mixed by ball milling at 600 rpm for 1 h. The weight ratio of ball to powder was 25 to 1. For the SPANI sample, the mixture was further heated at 300 °C for 10 h in a crucible with a heating rate of 100 ^o^C/h. The sulfur content in the SPANI sample was calculated by the weight lost upon heat treatment and was determined to be ~ 34 %. The cathode electrode was prepared by casting 90 wt% active material (S/PANI or SPANI) and 10 wt% CMC (Carboxymethyl Cellulose) / SBR (Styrene Butadiene Rubber) (w/w 2:1) in a solvent mixture of H_2_O and isopropanol. The slurry was then coated onto one side of carbon-coated Al foil, which was then dried at 40 ^o^C inside a vacuum oven. The loading mass of the active material for S/PANI or SPANI coin cell was ≈ 0.6−0.8 mg cm^−2^ and ≈ 0.3−0.4 mg cm^−2^, respectively. The electrodes were used directly after coating and drying without an additional calendaring/pressing process. Therefore, the electrode compaction density was not specifically optimized or quantified. The Ca anodes were obtained by a pressing and rolling process of a Ca chunk into Ca foil with a thickness of 100 μm and then punching out into proper disks with a diameter of 13 mm.^[2]^ This process was done in ambient atmosphere. Before being used as a Ca anode, these Ca disks were polished carefully in a glovebox. The carbon-coated separator was prepared according to our previous report.^[3]^ Typically, 90 wt% activated carbon filter (ACF) powder and 10 wt% Poly(vinylidenfluorid-co-hexafluorpropylen) (average Mw 400,000) in cyrene solution were mixed as a slurry. Subsequently, the homogeneous slurry was cast onto one side of the Celgard separator (Celgard 2325, PP/PE/PP, 25 µm thick). The dried ACF@Celgard separator was punched into disks with a diameter of 16 mm. The mass loading of the ACF was ≈ 0.1−0.2 mg cm^−2^.

***Electrochemical measurement*** The construction of the electrochemical cells was carried out in the argon-filled glove box. Symmetric Ca||Ca and asymmetric Ca||carbon-coated aluminium (ccAl) 2032 coin cells were employed to evaluate the cycling stability and cycle life span of the electrolyte. Symmetric Ca||Ca cells were assembled by using Ca disks as both working and counter electrodes. Asymmetric Ca||ccAl coin cells were assembled by using ccAl foil (Ø 12 mm) and Ca disks as both working and counter electrodes. Two-layer borosilicate glass fiber separators (GF/C from Whatman, Ø 18 mm) were used as the separator in the Ca||Ca and Ca||ccAl cell. The GF/C glass fiber separator was vacuum-dried at 230 °C overnight before use. The Ca−S batteries were assembled using 2032 coin cells with the S/PANI (or SPANI) cathode, a two-layer separator containing borosilicate glass fiber separators toward the Ca anode and carbon-coated Celgard 2325 separator toward the S/PANI cathode, and a Ca disc as the anode. Each cell was supplemented with 90 μL electrolyte. The electrolyte-to-sulfur (E/S) ratio of the S/PANI or SPANI coin cell was ≈ 99−132 μL mg^−1^ and ≈ 200−264 μL mg^−1^, respectively. For the high-sulfur-loading coin cell (≈ 3 mg cm^−2^), the E/S ratio was approximately 29 μL mg^−1^. The cells were placed in an incubator to maintain a constant temperature of 25 ± 0.1 °C. Cyclic voltammetry (CV) and galvanostatic cycling performance of the coin cells were investigated using a Biologic VMP-3 potentiostat.

***Pouch cell*** Pouch cells were assembled using cathode electrode sheets with dimensions of 30 × 50 mm^2^. For the Na-CaBhfip/DME pouch cell and the CaBhfip/DME pouch cell, S/PANI (S: 50 wt%) and S/KB (S: 45 wt%) were employed as the cathode materials, respectively. The sulfur loadings were 0.5 mg cm^−2^ for the Na-CaBhfip/DME pouch cell and 1 mg cm^−2^ for the CaBhfip/DME pouch cell. On the anode side, calcium foil with a thickness of 250 μm was punched into sheets with dimensions of 32 × 52 mm^2^. A carbon-coated Celgard 2325 separator, together with an additional borosilicate glass-fiber layer (53 × 60 mm^2^), was placed between the electrodes. The electrolyte volumes used were 1.5 mL for the Na-CaBhfip/DME pouch cell and 0.75 mL for the CaBhfip/DME pouch cell, respectively. The E/S ratio of the Na-CaBhfip/DME pouch cell and the CaBhfip/DME pouch cell was 200 μL mg^−1^ and 50 μL mg^−1^, respectively.

***Physicochemical characterizations*** FT-IR spectra were recorded with a Spectrum Two FT-IR Spectrometer (PerkinElmer) using the ATR mode. Measurements were collected in the range between 4000 and 400 cm^−1^ at room temperature. The morphology of the samples and FIB cross-sections were investigated using a Zeiss Crossbeam 350 with an EDAX Ocatane Super EDX System and a Zeiss Crossbeam 550 with an Oxford X-Max 150 EDX System at room temperature. SEM images were taken primarily at 3 kV. FIB cutting was performed with a gallium ion beam voltage of 30 kV. The current used for FIB cutting was 15 nA. For polishing, a current of 100 pA was used. X-ray photoelectron spectroscopy (XPS) measurements were carried out in a Specs XPS system. The spectra were acquired using monochromatic Al Kα radiation (1486.6 eV) and a pass energy of 30 eV at the analyzer for the high resolution spectra. The C 1*s* line of adventitious C on the electrode surface with a binding energy of 284.8 eV was used as a binding energy reference. CasaXPS was used for peak fit applying Shirley-type backgrounds and peaks with Gaussian-Lorentzian peak shape (GL30). For the peak doublets in the S *2p* and Ca *2p* regions, the expected intensity ratio (2:1) and spin-orbit splits (1.18 eV for S *2p* and 3.55 eV for Ca *2p*) were inserted.^[4]^

***DFT calculation*** DFT-calculations were performed using the Vienna *ab-initio* simulation package (VASP) version 6.4.2, which utilizes the projector-augmented wave (PAW) method. These calculations employed the Generalized Gradient Approximation (GGA) with the Perdew-Burke-Enzerhof (PBE) functional. A plane wave energy cutoff of 500 eV as well as a Gaussian-type smearing with a width of 0.05 eV were utilized. The wavefunctions were optimized to an accuracy of 10^–5^ eV and geometries were relaxed until forces were below 0.01 eV/Å. Surface calculations were performed on a single graphene layer or five layers for Na_2_S with 15 Å of vacuum and a 5×5×1 Monkhorst–Pack ***k***-point grid. Adsorbates were placed on the (110) surface of Na_2_S, which was found to be the most stable of the low-index surfaces of Na_2_S in our calculations. Adsorption energies were calculated as $E_{\mathrm{ads}}=E_{\mathrm{Ca}S_{n}+\mathrm{surface}}-E_{\mathrm{surface}}-E_{\mathrm{Ca}S_{n}}$, formation energies as $E_{f}=E_{\mathrm{Ca}S_{n}+surface}-E_{\mathrm{surface}}-E_{Ca,bulk}-\frac{n}{8}E_{S_{8}}$.

**Figures:**

**Figure S1.** Cycling performance of symmetric Ca||Ca coin cells at a current density of 0.1 mA cm^−2^ using modified electrolytes containing different concentrations of Na[B(hfip)_4_] in 0.3 M Ca[B(hfip)_4_]_2_/DME.

**Figure S2.** SEM image of Ca metal at the plated side after cycling in the symmetric Ca||Ca coin cell with (a) 0.05 M Na[B(hfip)_4_] in 0.3 M Ca[B(hfip)_4_]_2_/DME and (b) 0.3 M Na[B(hfip)_4_] in 0.3 M CaCaBhfip/DME electrolyte.

**Figure S3.** LSVs of CaBhfip/DME and Na-CaBhfip/DME using stainless steel as the WE and Ca as the RE and CE at a scan rate of 1 mV s^−1^.

**Figure S4.** Digital images of the Ca metal after cycling in symmetric Ca||Ca coin cells with CaBhfip/DME (left) and Na-CaBhfip/DME electrolyte (right).

**Figure S5.** (a) Cross-sectional FIB−SEM image and (b) the corresponding C, Ca, Na, F EDS element mapping images of the Ca metal at the plated side after cycling in the symmetric Ca||Ca coin cell with CaBhfip/DME electrolyte.

**Figure S6.** SEM image of Ca metal at the stripped side after cycling in the symmetric Ca||Ca coin cell with CaBhfip/DME electrolyte.

**Figure S7.** FT−IR spectrum of the PANI and S/PANI sample.

**Figure S8.** Electrochemical performance of PANI electrode. (a) CV curves at 0.1 mV s^−1^, (b) GCD profiles of the Ca||PANI coin cell with the CaBhfip/DME electrolyte at 300 mA g^−1^. The PANI electrode was prepared by casting 80 wt% PANI, 10 wt% Super P, and 10 wt% PVDF in 1-Methyl-2-pyrrolidone. The slurry was then coated onto one side of carbon-coated Al foil, which was then dried at 60 ^o^C inside a vacuum oven. The loading mass of the PANI was ≈ 0.4 mg cm^−2^.

**Figure S9.** Galvanostatic charge/discharge profiles of the Ca|Na-CaBhfip/DME|S/PANI coin cell at 0.1C in selected cycles. Inset: GCD profile at the 80^th^ cycle, showing the preserved discharge plateau at ~2.2 V.

**Figure S10.** GCD profiles of the Ca|0.15 M Na[B(hfip)_4_]|PANI coin cell at 300 mA g^−1^.

**Figure S11.** (a) Cycling performance and (b) GCD profiles of the Ca|Na-CaBhfip/DME|S/PANI coin cell at 0.1C.

**Figure S12.** High resolution S 2*p* XPS spectrum of the pristine S/PANI cathode.

**Figure S13.** High resolution Ca 2*p* XPS spectra of the pristine S/PANI cathode and after discharge to 1.90 V, 1.25 V, 0.50 V, and charge to 3.5 V at 0.1C.

**Figure S14.** High-resolution Na 1*s* XPS spectrum of the pristine S/PANI electrode.

**Figure S15.** Postmortem SEM and EDS analysis of the cathode.

**Figure S16.** Postmortem EDS quantitative analysis of the element ratio of the cathode at different states.

**Figure S17**. Optimized configurations for the binding of the S_8_ and representative Ca polysulfide species (CaS, CaS_2_, CaS_4_, CaS_6_, and CaS_8_) to graphene.

**Figure S18**. Optimized configurations for the binding of the S_8_ and representative Ca polysulfide species (CaS, CaS_2_, CaS_4_, CaS_6_, and CaS_8_) to Na_2_S.

**Figure S19.** Cycling performance (a) and GCD profiles (b) of the Ca−S pouch cell with the CaBhfip/DME electrolyte.

**Figure S20.** Coulombic efficiency of the Ca−S pouch cell with the Na-CaBhfip/DME electrolytes at 0.1C.

**Figure S21.** FTIR spectrum of the PANI and SPANI sample.

**Figure S22.** GCD profiles of the Ca||SPANI coin cell with CaBhfip/DME electrolyte at 0.1C.

**Table S1**. Electrochemical performance comparison of reported Ca−S Batteries with different electrolyte systems.

| Cathode | Sulfur loading  (mg cm^-2^) | Electrolyte | Anode | Current density  (mA g^−1^) | Plateau  (V) | Initial Capacity  (mAh g^−1^) | Final capacity  (mAh g^−1^) | Cycle number | Ref. |
| --- | --- | --- | --- | --- | --- | --- | --- | --- | --- |
| S@C NS | ~0.8 | 0.2M LiPF_6_ and 0.06M Ca(BF_4_)_2_ | Ca | 50 | ~2.0 | 824 | 395 | 150 | ^[5]^ |
| S/CNF | 2.0 | 0.2M Ca(CF_3_SO_3_)_2_ and 0.5 M LiCF_3_SO_3_ | Ca | 167.5 | ~1.4 | ≈800 | ≈300 | 20 | ^[6]^ |
| S/C | 0.8−1 | 0.5M Ca[B(hfip)_4_]_2_/DME | Ca | 167.5 | ~2.1 | ~760 | ~200 | 15 | ^[7]^ |
| S@C | 0.5−0.8 | 0.3M Ca(PF6)_2_ and 0.6M NaPF_6_ | Na | 167.5 | ~1.0 | 947.4 | 792 | 200 | ^[8]^ |
| S/KB | N/A | 0.25M Ca[B(hfip)_4_]_2_ | Ca | N/A | ~2.2 | 760 | 120 | 15 | ^[2]^ |
| S/PANI | 0.6−0.8 | 0.3M Ca[B(hfip)_4_]_2_ and 0.15M Na[B(hfip)_4_] | Ca | 167.5 | ~2.2 | 860 | 92 | 80 | This work |
|  | 3 |  |  |  | ~2.1 | 409.4 | 73.5 | 50 |  |
| SPANI | 0.3−0.4 |  |  |  | ~2.2 | 393.1 | 349.2 | 15 |  |

| **Table S2. Element content (atomic %) of the cycled S/PANI cathode in XPS spectra.** | | | | | | | | |
| --- | --- | --- | --- | --- | --- | --- | --- | --- |
|  | C 1*s* | O 1*s* | F 1*s* | S 2*p* | B 1*s* | Cl 2*p* | Ca 2*p* | Na 1*s* |
| Pristine | 74.18% | 17.89% | 0.00% | 4.65% | 0.00% | 0.00% | 0.00% | 1.52% |
| D-1.9V | 46.81% | 16.06% | 29.27% | 0.47% | 2.65% | 0.35% | 2.39% | 1.99% |
| D-1.25V | 46.91% | 17.28% | 28.15% | 0.42% | 2.43% | 0.34% | 2.55% | 1.93% |
| D-0.5V | 46.08% | 22.39% | 20.23% | 0.85% | 4.61% | 0.46% | 3.89% | 1.49% |
| C-3.5V | 43.47% | 18.96% | 28.19% | 0.98% | 4.60% | 0.25% | 3.14% | 0.41% |

| **Table S3. Normalized element content (Atomic%) of the cycled S/PANI cathode in XPS spectra.** | | | | | | | | |
| --- | --- | --- | --- | --- | --- | --- | --- | --- |
|  | C 1*s* | O 1*s* | F 1*s* | S 2*p* | B 1*s* | Cl 2*p* | Ca 2*p* | Na 1*s* |
| Pristine | 1 | 24.12% | 0.00% | 6.27% | 0.00% | 0.00% | 0.00% | 2.05% |
| D-1.9V | 1 | 34.32% | 62.54% | 1.00% | 5.67% | 0.75% | 5.11% | 4.26% |
| D-1.25V | 1 | 36.83% | 60.01% | 0.89% | 5.17% | 0.73% | 5.43% | 4.11% |
| D-0.5V | 1 | 48.59% | 43.89% | 1.84% | 10.01% | 0.99% | 8.45% | 3.24% |
| C-3.5V | 1 | 43.61% | 64.84% | 2.26% | 10.57% | 0.58% | 7.23% | 0.95% |

**Reference:**

[1]	a) Z. Li, O. Fuhr, M. Fichtner, Z. Zhao-Karger, *Energy Environ. Sci.* **2019**, *12*, 3496-3501; b) A. Roy, M. Sotoudeh, S. Dinda, Y. Tang, C. Kubel, A. Gross, Z. Zhao-Karger, M. Fichtner, Z. Li, *Nat. Commun.* **2024**, *15*, 492.

[2]	Z. Li, B. P. Vinayan, T. Diemant, R. J. Behm, M. Fichtner, Z. Zhao-Karger, *Small* **2020**, *16*, e2001806.

[3]	L. Wang, P. Jankowski, C. Njel, W. Bauer, Z. Li, Z. Meng, B. Dasari, T. Vegge, J. M. G. Lastra, Z. Zhao-Karger, M. Fichtner, *Adv. Sci.* **2022**, *9*, e2104605.

[4]	J. F. Moulder, W. F. Stickle, P. E. Sobol, K. D. Bomben, Handbook of X-ray Photoelectron Spectroscopy, Perkin Elmer Corp., Eden Prairie (USA), 1992.

[5]	S. Wu, Z. Jiang, C. Wu, H. Li, J. Huang, N. Li, S. Huang, *Adv. Funct. Mater.* **2024**, *34*, 2313441.

[6]	X. Yu, M. J. Boyer, G. S. Hwang, A. Manthiram, *Adv. Energy Mater.* **2019**, *9*, 1803794.

[7]	A. Scafuri, R. Berthelot, K. Pirnat, A. Vizintin, J. Bitenc, G. Aquilanti, D. Foix, R. Dedryvère, I. Arčon, R. Dominko, L. Stievano, *Chem. Mater.* **2020**, *32*, 8266-8275.

[8]	D. Zhou, X. Tang, X. Zhang, F. Zhang, J. Wu, F. Kang, B. Li, G. Wang, *Nano Lett.* **2021**, *21*, 3548-3556.
